# Merkel cell polyomavirus detected in head and neck carcinomas from Chile

**DOI:** 10.1186/s13027-020-0276-z

**Published:** 2020-01-28

**Authors:** Juan P. Muñoz, Rancés Blanco, Julio C. Osorio, Carolina Oliva, María José Diaz, Diego Carrillo-Beltrán, Rebeca Aguayo, Andrés Castillo, Julio C. Tapia, Gloria M. Calaf, Aldo Gaggero, Francisco Aguayo

**Affiliations:** 1 Instituto de Alta Investigación (IAI), Universidad de Tarapaca, Arica, Chile; 2 Programa de Virología, Instituto de Ciencias Biomédicas (ICBM), Faculty of Medicine, Universidad de Chile, Santiago, Chile; 3 Centro de Salud Familiar “El Roble”, Municipalidad de La Pintana, Santiago, Chile; 4 Department of Biology, Faculty of Natural and Exact Sciences at Universidad del Valle, Cali, Colombia; 5 Departamento de Oncología Básico Clínica, Faculty of Medicine, University of Chile, Santiago, Chile; 6 Center for Radiological Research, Columbia University Medical Center, New York, NY USA; 7 Advanced Center for Chronic diseases (ACCDiS), Universidad de Chile, Santiago, Chile

**Keywords:** Cancer, Head and neck, Polyomavirus

## Abstract

**Background:**

The role of human polyomaviruses (HPyVs) in epithelial tumors such as head and neck carcinomas (HNSCCs) including oral and oropharyngeal carcinomas has not been established. In this study, we evaluated for the first time the presence of Merkel cell polyomavirus (MCPyV), BK human polyomavirus (BKPyV), and JC human polyomavirus (JCPyV) in HNSCCs from Chilean subjects.

**Methods:**

One hundred and twenty HNSCCs were analyzed for the presence of MCPyV, BKPyV and JCPyV using real-time polymerase chain reaction procedures. In addition, 54 oral brushes from age- and sex-paired subjects were analyzed.

**Results:**

Of the total of 120 HNSCCs, 15 were positive for MCPyV (12.5%). Only one case was positive for BKPyV (0.8%) and none for JCPyV (0%). In subjects without cancer, only one case (1.8%) resulted positive for MCPyV and none for JCPyV and BKPyV. MCPyV was associated with HNSCCs (*p* = 0.0239; OR = 7.571; 95% CI: 1.192–81.46). No association was found between age (*p* = 0.1996), gender (*p* = 0.7111) or differentiation status (*p* > 0.9999) and MCPyV presence in HNSCCs.

**Conclusions:**

MCPyVs were detected in HNSCCs from Chilean patients and were not detected in oral brushes from patients without cancer. More studies are warranted for defining an etiological role and clinical/molecular consequences of these viruses in HNSCCs.

## Introduction

Human polyomaviruses (HPyVs) are small DNA viruses classified into the Polyomaviridae family and are widely distributed in human population [1]. BKPyV and JCPyV were discovered in 1971. In the last 15 years, mainly due to the development of new high-throughput sequencing techniques, other twelve HPyVs have been discovered (MCPyV, KIPyV, WUPyV, TSPyV, HPyV6, HPyV7, HPyV9, HPyV10, HPyV12, STLPyV, NJPyV and LIPyV) [2, 3]. HPyV infection usually occurs early in life through a fecal-oral transmission mechanism and persists for the lifetime of the infected subject. In natural hosts, these viruses establish a productive infection, although in heterologous non-permissive hosts, the virus establishes latency with potential integration into the host genome [2]. HPyVs are very ubiquitous in nature and their association with some pathologies, cancer in particular, is under intensive investigation [4]. All members of the HPyV family encode the Large and small T-antigens that exhibit variable oncogenic potential in in vitro and animal models oncogenic protein T-antigen. In some cases, these proteins can inactivate p53, members of the pRb protein family and other cellular factors diminishing apoptosis and deregulating the cell cycle [5]. In addition, T-antigen interacts with beta-catenin, enhancing the transcription of the c-myc promoter [6]. However, out of the HPyV family, only MCPyV has been causally associated with human cancer, specifically Merkel cell carcinoma, a very rare skin cancer with a neuroendocrine origin [7, 8]. The role of other HPyVs such as BKPyV and JCPyV in human cancer remains highly controversial and unclear [9–11].

Head and neck cancers (HNCs) comprise different malignancies which include lip, oral cavity, nasopharynx, oropharynx, hypopharynx, pharynx and larynx cancer [12]. The worldwide incidence of these malignant tumors is more than 550,000 cases with 300,000 deaths per year with a male to female ratio that ranges from 2:1 to 4:1 [13]. Approximately 90% of HNCs are squamous cell carcinomas (HNSCCs), the sixth leading cancer by incidence worldwide [12]. In Chile, it has been estimated that lip and oral cancer incidence is 1.3 cases per 100,000 inhabitants; oropharynx and hypopharynx is 0.5 cases per 100,000 inhabitants, and larynx is 1.2 cases per 100,000 inhabitants [14].

HNSCC is strongly associated with certain environmental and lifestyle risk factors such as tobacco smoking and alcohol consumption [15]. In addition, infection with high-risk human papillomavirus (HR-HPV), especially HPV type 16, is a risk factor for oropharyngeal tumors that involve the tonsils or the base of the tongue [16], while infection with the Epstein-Barr virus (EBV) is a risk factor for nasopharyngeal and salivary gland cancers [17]. In fact, our group previously reported that 11% of oral carcinomas were positive for HR-HPV in Chilean patients. Moreover, the most frequent HPV genotype found in this study was HPV16, a high-risk HPV type [18]. It is unknown whether other DNA viruses are involved in the development of HNSCC. Therefore, in this study we evaluated the prevalence of MCPyV, BKPyV and JCPyV in oral and oropharyngeal carcinomas from Chilean patients for the first time.

## Materials and methods

### Clinical specimens

We conducted an analysis of 135 oral and oropharyngeal carcinomas from Chilean patients and 54 oral brushes from sex- and age-paired healthy Chilean subjects. Tumor samples were collected at Instituto Nacional del Cancer (INC) “Caupolicán Pardo”; Facultad de Odontología, Universidad de Chile; and “San José” Hospital (Santiago, Chile). All cases were confirmed by histological analyses, of which 99 corresponded to formalin-fixed and paraffin-embedded (FFPE) specimens obtained between 2003 and 2014 and 36 frozen tissues stored at − 80 °C, obtained between 2014 and 2016. The 54 oral brushing samples were collected during 2016, from patients without neoplasia who were treated for minor dental problems in CESFAM “El Roble” (La Pintana, Santiago). The patients provided their written informed consent to participate in the study, which was approved by the respective ethics committee of each institution. Frozen tissues with a weight < 1 g and paraffin tissue sections with < 10% of tumor cells were excluded. This study was approved by the Ethical Committee Board of the Faculty of Medicine, University of Chile (Number 071–2011).

### DNA extraction

Paraffin tissue sections were incubated with digestion buffer (10 mM Tris–HCl pH 7.4, 0.5 mg/mL proteinase K and 0.4% Tween 20) at 37 °C overnight. Afterwards, the samples were incubated for 10 min at 95 °C, centrifuged for 2 min at 14,000 rpm and immediately placed on ice. After centrifugation, the aqueous phase was transferred to a new tube for analysis.

### Real-time polymerase chain reaction (qPCR)

BKPyV detection was conducted using qPCR with an intercalating dye format as previously reported [19]. Briefly, a 127 bp fragment of the BKPyV genome was amplified with the following BK127-F and BK127-R primers: BK127-F: 5′-GCA GCT CCC AAA AAG CCA AA-3′ and BK127-R: 5′-CTG GGT TTA GGA AGC ATT CTA-3′. The 20 μL final volume reactions contained 1X LightCycler® FastStart DNA Master SYBR Green I (Roche Diagnostics, Indianapolis, IN, USA), 0.5 μM of each primer, 3.0 μM MgCl2 and 5 μL of DNA template. The PCR reaction was run for 40 cycles as follows: denaturation at 94 °C for 10 s, annealing at 62 °C for 10 s and extension at 72 °C for 10 s, with an initial denaturation at 95 °C for 15 min and final extension at 72 °C for 10 min. The amplification products were subjected to a melting profile and the specific product was characterized according to its Tm. JCPyV was detected using qPCR with JE3 primers and a JE3 probe set (JE3-(Mad-1) F: 5′-ATG TTT GCC AGT GAT GAT GAA AA-3′, JE3-(Mad-1) R: 5′-GGA AAG TCT TTA GGG TCT TCT ACC TTT-3′ and JE3-(Mad-1) Probe 5′-FAM-AGG ATC CCA ACA CTC TAC CCC ACC TAA AAA GA-BHQ1–3′) according to a previously described protocol [20] The PCR reaction contained 1X Brilliant® II QRT-PCR Master Mix (Agilent Technologies Santa Clara, CA, USA), 0.5 μM of each primer, 0.15 μM of probe and 5 μL of DNA in a total volume of 25 μL. The amplification conditions were as follows: 95 °C for 10 min, followed by 50 cycles with denaturation at 95 °C for 15 s and annealing/extension at 60 °C for 1 min. The MCPyV detection was conducted using qPCR with a TaqMan probe (FAM-TCCTTCTCAGCGTCCCAGGCTTCA-TAMRA) in LightCycler® 2.0 (Roche Diagnostics, Indianapolis, IN, USA), as previously reported. Briefly, a 146 fragment of the TAg gene was amplified with the primers: LT1-F: 5′- CCACAGCCAGAGCTCTTCCT-3′ and LT1-R: 5′- TGGTGGTCTCCTCTCTGCTACTG-3′. The PCR reaction contained 1X TaqMan Universal PCR Master Mix (Bioline Ltd., London, UK), 0.9 μM of each primer and 0.5 μM of TaqMan probe and 5 μL of DNA in a total volume of 20 μL. The PCR reaction was run as follows: 50 °C for 2 min, 95 °C for 10 min, followed by 45 cycles with denaturation at 95 °C for 5 s, annealing at 60 °C for 1 min and extension at 40 °C for 30 s.

### Statistical analysis

For categorical variables, the frequency tables were analyzed using the χ2-test or Fisher’s exact statistics. Odds ratio (OR) and their 95% confidence intervals (95% CI) were calculated where appropriate, using the exact method. All statistical tests were performed as two-sided and considered significant at *p*-value < 0.05. Statistical analyses were run using the GraphPad Prism 8 software.

## Results

### Clinical-pathological features

We analyzed the presence of MCPyV, BKPyV and JCPyV in Chilean patients diagnosed with oral cavity and oropharyngeal carcinomas through a retrospective study. Of the 99 FFPE samples, 15 were excluded because they tested negative for the β-globin fragment (internal control) or not enough tissue material was available. The 36 frozen tissue specimens amplified for the same fragment. Table 1 shows the clinicopathological characteristics of the 120 patients included in this study. The 51.4% of patients corresponds to women and 48.6% to men, with an average age of 63.6 years (range 30–97 years). The majority of tumor samples (55.9%) were classified as well differentiated grade.

**Table 1:** Clinicopathological features of HNSCC patients

Variable	HNSCC cases
	(*n* = 120)
	*number (percentages)*
Gender	
Female	37 (51.4)
Male	35 (48.6)
Unknown	48
Age	
< 65 yr	31 (47.7)
≥ 65 yr	34 (52.3)
Unknown	55
Average (yr)	63.6
Range (yr)	30–97
Tumor differentiation	
Well	19 (55.9)
Moderate	12 (35.3)
Poorly	3 (8.8)
Unknown	86

Legend. *HNSCC* Head and Neck squamous cell carcinoma; *yr* years

### HPyVs presence in head and neck carcinomas

Fifteen of the HNSCCs were positive for MCPyV (12.5%), of which 9/15 (60.0%) and 6/15 (40.0%) correspond to FFPE and frozen samples, respectively. Additionally, 1(1,8%) HNSCC was positive for JCPyV and none for BKPyV. In contrast, MCPyV was only detected in 1/54 (1.8%) of healthy patients. JCPyV and BKPyV were negative for this group (Table 2). The difference of MCPyV presence between HNSCCs and oral brushes was statistically significant (*p* = 0.0239; OR = 7.571; 95% CI: 1.192–81.46) (Table 3). Among positive cases, the infection with MCPyV was mostly detected in women (5/8 cases; 62.5%), in patients ≥65 years (5/6; 83.3%) as well as in tumors displaying moderate and well differentiated grades (2/2; 100%). However, no statistically significant differences were found between age (*p* = 0.1996), gender (*p* = 0.7111) or tumor differentiation (*p* > 0.9999) and MCPyV presence in HNSCCs.

**Table 2:** Presence of HPyVs in HNSCCS and oral brushes from Chilean patients

Cases (total)	MCPyV	JCPyV	BKPyV
Oral brushes (54)	1 (1.8)	0 (0)	0 (0)
HNSCCS (120)	15 (12.5)	1 (0.8)	0 (0)

Legend. *HNSCC* Head and Neck squamous cell carcinoma; *MCPyV* Merkel cell polyomavirus; *JCPyV* JC polyomavirus; *BKPyV* BK polyomavirus

**Table 3:** MCPyV in HNSCCS and oral brushes from Chilean patients

Variable	MCPyV		OR (95% CI)	
	Positive	Negative		*P* - value
	(*n* = 16)	(*n* = 158)		
	*number (percentage)*			
Oral brushes	1 (1.85)	53 (98.15)	7.571 (1.192–81.46)	0.0239
HNSCCs	15 (12.5)	105 (87.5)		

Legend. *OR* Odds Ratio; *CI* Confidence Interval

## Discussion

In this study, we found that MCPyV is frequently present in HNSCCs from Chilean patients. Our data report the occurrence of MCPyV infection in 12.5% of HNSCCs samples. In contrast, the presence of MCPyV was only detected in 1.8% of healthy subjects. Similarly, *Mohebbi* et al. published the MCPyV positivity in 16% of HNSCCs from Iranian patients vs. 2% in the control group [21]. However, *Tanio* et al. reported a low MCPyV prevalence of 4.0% in oral cavity carcinoma from Japanese patients [22], while *Hamiter* et al. described that 28.6% of squamous cell carcinoma of the tongue were positive for MCPyV DNA sequences in North American population [23]. Of interest, in the Czech population, MCPyV was positive in 35.7% of squamous cell tonsillar carcinomas and in 10.2% of non-malignant tissues [24]. No associations of MCPyV and clinicopathological features of HNSCC patients were evidenced. In previous studies, no significant difference between MCPyV positivity with age, gender, type of HNSCC, and cancer stages was reported. However, MCPyV DNA viral load was associated with advanced stages of HNSCCs [21]. On the contrary, the presence of MCPyV in oral tongue SCC was associated with lower recurrence rates [23]. In this regard, the relation of MCPyV presence in HNSCCs with the biological behavior of these malignancies remains controversial. A limitation of our study was the extensive lack of clinical information. This restricted the establishment of better associations between the presence of MCPyV and clinicopathological features of cancer patients, which need to be addressed in further studies. In the case of the virus BKPyV, only one infected sample (0.8%) of HNSCCs was detected, similar to a previous report conducted by *Poluschkin* et al. in which 1.2% of oral carcinoma cases from Finnish patients was considered positive [25]. In contrast, two different studies conducted in Lublin (Poland) showed the presence of BKPyV DNA in 18.5 and 17.7% of patients with oropharyngeal and oral carcinomas, respectively, and only in 3.3% of the control group [26]. This fact encouraged consideration of the role of BKV virus in the development of oral squamous cell carcinoma [26, 27]. Moreover, in the same population, *Drop* et al. found BKPyV DNA in 17.2% (11/64) of HNSCCs including oropharynx (14.3%), larynx (18.2%) and oral cavity (21.4%) [28], evidencing a preferential presence of BKPyV in HNSCCs patients from this country. In addition, we found that oral and oropharyngeal samples did not show JCPyV DNA amplification, similar to a previous report conducted by *Polz* et al. in oral carcinomas [26]. Nevertheless, *Poluschkin* et al. published infection with JCPyV in 37% of HNSCCs, including lip (80%), larynx (67%) and oral cavity (59%), but not in oropharyngeal carcinomas [25]. *Monaco* et al. reported the presence of JCPyV DNA in about 35% of tonsil tissue from both immunocompetent children and healthy adult donors, providing evidence concerning the site of infection [29]. Interestingly, *Kutsuna* et al. found more than 200 copies/μg of JCV-targeting T antigen in 92.3% of tongue squamous carcinomas, which was significantly increased when compared with dysplastic tongue epithelium and glossitis [30], suggesting a potential association of JCPyV load with this type of cancer. In the present study, the number of samples infected with MCPyV, BKPyV and JCPyV in HNSCCs from Chilean patients was generally found to be low when compared with other populations around the world. Regarding MCPyV, it is the only polyomavirus clearly associated with a human cancer, Merkel cell carcinoma (MCC), which mainly occurs in Caucasians as well as in populations located in equatorial proximity [7]. Concerning BKPyV and JCPyV, they were associated with prostatic and colorectal cancer, respectively [31, 32]. Of note, the existence of various genotypes linked to the geographical origin of the infected individuals has been reported [33]. These population differences are important because of the differential risk of developing cancer. In this sense, the evaluation of other risk factors implicated in head and neck carcinogenesis are of great significance.

The clinical and molecular consequences of MCPyV presence in HNSCCS from Chilean patients are unknown. Previously, we reported the presence of high-risk HPV in a subset of oral carcinomas from Chilean patients [18]. Thus, a possible collaboration between HPV and MCPyV in head and neck cancer is an interesting point that needs to be investigated in the future, as previously reported in laryngeal cancer [34].

To summarize, in this study we evaluated the prevalence of MCPyV, BKPyV and JCPyV in HNSCCs from Chilean patients for the first time. MCPyV was increased in HNSCCs when compared with BKPyV and JCPyV, suggesting an association of MCPyV infection with these malignant tumors. However, no relation between MCPyV infection and age, gender, tumor localization or differentiation status was evidenced.

## Conclusions

MCPyV is present in HNSCCs from Chilean patients and is almost absent in oral brushes from healthy subjects. Further studies are strongly necessary in order to elucidate a potential etiological role for this virus in the development and/or progression of this cancer.

## Data Availability

The datasets used and/or analyzed during the current study are available from the corresponding author on reasonable request.

## Abbreviations

BKPyV: BK human polyomavirus
CI: Confidence intervals
EBV: Epstein-Barr virus
FFPE: Formalin-fixed and paraffin-embedded
HNCs: Head and neck cancers
HNSCCs: Head and neck carcinomas
HPyVs: Human polyomaviruses
HR-HPV: High-risk human papillomavirus
JCPyV: JC human polyomavirus
MCC: Merkel cell carcinoma
MCPyV: Merkel cell polyomavirus
OR: Odds ratio
qPCR: Quantitative polymerase chain reaction
